# Benign Cutaneous Cysts: A Comprehensive Analysis of 1160 cases

**DOI:** 10.7759/cureus.45548

**Published:** 2023-09-19

**Authors:** Anila Chughtai, Muhammad Moseeb Ali Hashim, Rashida Saleem, Ghazi Zafar, Rafeya Yasin, Omar Chughtai, Akhtar S Chughtai, Asma Zafar

**Affiliations:** 1 Histopathology, Chughtai Institute of Pathology, Lahore, PAK

**Keywords:** steatocystoma, comedones, benign cutaneous cysts, hidrocystoma, trichilemmal cyst, dermoid cyst, epidermal inclusion cyst

## Abstract

Background

Cysts are common skin abnormalities that are mostly benign; however, sometimes malignant lesions may present clinically as cystic manifestations. Benign cutaneous cysts can be of different morphological types and their diagnosis relies on histological evaluations. The most common mode of treatment is surgical excision, which is curative.

Methodology

This is a retrospective cross-sectional study conducted at the Department of Histopathology, Chughtai Institute of Pathology, Lahore, Pakistan from 1st January 2020 to 31st December 2022. Non-probability consecutive sampling was done, and all the cases of benign cutaneous cysts were included. All cases were microscopically reviewed by two histopathologists, and variables like age, gender, site of the lesion, and histological diagnosis were noted. The data were analyzed using IBM SPSS Statistics for Windows, Version 29 (Released 2022; IBM Corp., Armonk, New York, United States).

Results

A total of 1160 recorded cases of benign cutaneous cysts were included. Overall gender distribution revealed males (n=489, 42.1%) and females (n=671, 57.8%). The age range was 3 to 91 years with a mean age of 37.56 ± 16.05 years. The three most common cysts were epidermal inclusion cysts (74.3%), trichilemmal cysts (15.1%), and dermoid cysts (6.3%). Other cysts were uncommon including hidrocystoma (1.9%), steatocystoma (0.3%), verrucous cysts (0.3%), comedones (0.6%), hybrid cysts (0.2%), milia (0.3%), and vellus hair cysts (0.2%). The most common site was back (23.5%) for epidermal inclusion cysts, scalp (74.4%) for trichilemmal cysts, and eye (33.8%) for dermoid cysts.

Conclusion

Benign cutaneous cysts have a broad morphological spectrum with a wide age range. Epidermal inclusion cysts, trichilemmal cysts, dermoid cysts, and hidrocystoma account for the four most common types. For each of the other cyst type, the prevalence was under 1%. Female gender predominated in epidermal inclusion cysts, trichilemmal cysts, and dermoid cysts while male gender was common in other cysts. Overall majority of the cysts presented in the head and neck area.

## Introduction

Benign cutaneous cysts are prevalent skin abnormalities that can lead to aesthetic and medical issues, eliciting significant concern among both patients and their healthcare providers. Cysts are fluid-filled cavitary lesions, lined by different types of epithelium [1]. The cutaneous cysts are mostly benign; however, sometimes malignant lesions may also present as cystic lesions. Benign cutaneous cysts can be of various morphological types. These may present as either dermal or subcutaneous nodules [2]. These cysts are mostly painless; however, they can be painful if ruptured or infected. Surgical excision is the primary mode of treatment and is curative [3]. The diagnosis of cysts relies on the clinical characteristics of the lesions followed by histopathological examination to determine the exact morphological type and to exclude the possibility of malignancy [3-6]. To the best of our knowledge, there are no detailed studies related to benign cutaneous cysts conducted in the Pakistani population. The aim of this study was to assess the prevalence of different types of benign cutaneous cysts in the Pakistani population and describe their detailed clinical and pathological characteristic.

## Materials and methods

Study design

This is a retrospective cross-sectional study conducted at the Department of Histopathology, Chughtai Institute of Pathology, Lahore, Pakistan from 1st January 2020 to 31st December 2022. The study was conducted after approval by the institutional review board (CIP/IRB/1151). The non-probability consecutive sampling technique was applied.

Inclusion criteria

All the biopsy cases with a diagnosis of benign cutaneous cysts reported in the designated time window were included in the study.

Exclusion criteria

The cases with poor preservation due to fixation artifacts of the tissue were excluded from the study.

Data collection

The tissue slides for all the reported cases within the study period were retrieved from archives. These slides were prepared from paraffin embedded tissue blocks which were formed after routine tissue processing. The slides were stained with hematoxylin and eosin tissue stains. All the case slides were reviewed by two histopathologists with special interest in dermatopathology and findings were noted. The variables like age, gender, site of the lesion, and histological diagnosis were noted.

Statistical analysis

The data were analyzed using IBM SPSS Statistics for Windows, Version 29 (Released 2022; IBM Corp., Armonk, New York, United States).

## Results

A total of 1160 recorded cases of benign cutaneous cysts were included in the study. Overall gender distribution revealed males (n=489, 42.1%) and females (n=671, 57.8%). The age range was 3 to 91 years with a mean age of 37.56 ± 16.05 years. The most prevalent morphological type was epidermal inclusion cysts (n=862, 74.3%) followed by trichilemmal cysts (n=176, 15.1%), dermoid cysts (n=74, 6.3%), hidrocystoma (n=23, 1.9%), comedones (n=7, 0.6%), steatocystoma (n=4, 0.3%), verrucous cysts (n=4, 0.3%), milia (n=4, 0.3%), vellus hair cysts (n=3, 0.2%), and hybrid cysts (n=3, 0.2%) as shown in Table 1.

**Table 1:** Prevalence of different morphological types of benign cutaneous cysts

Cyst type	Frequency (n)	Percentage (%)
Epidermal inclusion cyst	862	74.3%
Trichilemmal cyst	176	15.1%
Dermoid cyst	74	6.3%
Hidrocystoma	23	1.9%
Comedones	7	0.6%
Steatocystoma	4	0.3%
Verrucous cyst	4	0.3%
Milia	4	0.3%
Vellus hair cyst	3	0.2%
Hybrid cyst	3	0.2%

For epidermal inclusion cysts, the majority of the cases presented in the fourth decade of life, and gender distribution revealed males (n=358, 41.5%) and females (n=504, 58.4%). Gender distribution and the most common age range for other cysts are shown in Table 2.

**Table 2:** Distribution of cysts according to gender predilection, most common age range, and most common location

Cyst type	Male	Female	Most common Age range (years)	Most common location
Epidermal inclusion cyst	358 (41.5%)	504 (58.4%)	31-40 (27.9%)	Back (23.5%)
Trichilemmal cyst	69 (39.2%)	107 (60.8%)	31-40 (31.3%)	Scalp (74.4%)
Dermoid cyst	35 (47.3%)	39 (52.7%)	11-29 (40.5%)	Eye (33.8%)
Hidrocystoma	12 (52.2%)	11 (47.8%)	31-50 (43.4%)	Eye (78.3%)
Comedone	7 (100%)	0	11-20 (57%)	Face (71.4%)
Steatocystoma	3 (75%)	1 (25%)	21-30 (50%)	Eye (75%)
Verrucous cyst	4 (100%)	0	41-50 (50%)	Scalp (75%)
Milia	3 (75%)	1 (25%)	21-30 (50%)	Face (75%)
Vellus hair cyst	3 (100%)	0	11-20 (100%)	Face (66%)
Hybrid cyst	1 (33%)	2 (66%)	21-30 (66%)	Back (66%)

The most common site of presentation is back (23.5%) in epidermal inclusion cysts, scalp (74.4%) in trichilemmal cysts, eye (33.8%) in dermoid cysts, eye (78.3%) in hidrocystoma, face (71.4%) in comedones, eye (75%) in steatocystoma, scalp (75%) in verrucous cysts, face (75%) in milia, face (66%) in vellus hair cysts, and back (66%) in hybrid cysts as depicted in Table 2.

## Discussion

**Figure 1:**
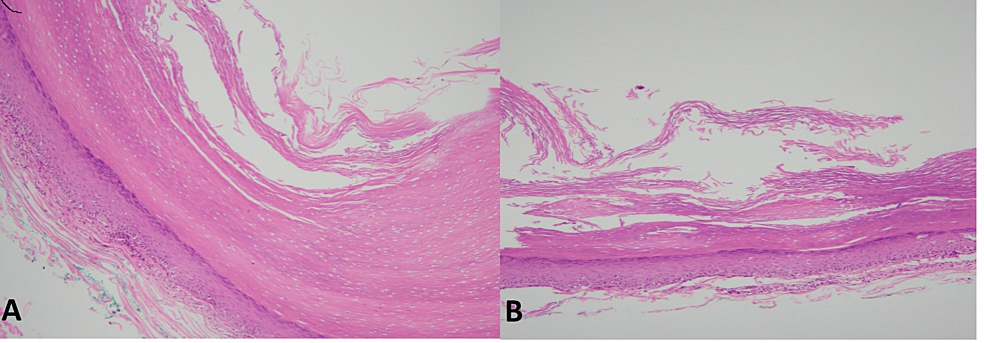
Epidermal inclusion cyst A and B: Cyst showing stratified squamous epithelium with an intact granular layer and flaky keratin in the lumen

Benign cutaneous cysts are one of the most common skin lesions encountered clinically. The benign cutaneous cysts have a broad morphological spectrum that correlates with the structures of origin, mainly including the epidermis, pilosebaceous units, and eccrine/apocrine sweat ducts [1,2]. A vast majority of cystic lesions of skin are benign; however, sometimes malignancies can perfectly imitate cystic clinical presentation necessitating histological evaluation [3-6]. The most common type of benign cutaneous cyst reported in the literature is the epidermal inclusion cyst (EIC) which correlates with our findings as it accounted for 74.3% of all the cysts included in this study [7]. It was more prevalent in females with the majority of cases presenting back in the fourth decade of life. These findings are discordant with the findings of Nigam et al. [8]. Histologically, it showed benign stratified squamous epithelium with an intact granular layer, flaky keratinous material within the luminal cavity, and associated inflammation in ruptured cases, shown in Figure 1 [3,9]. 

**Figure 2:**
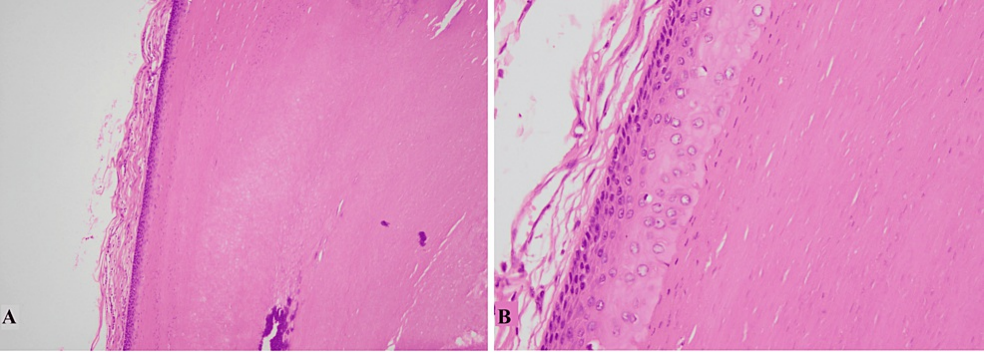
Trichilemmal cyst A: Low-power view of the cyst with abrupt trichilemmal type of keratinization. B: High-power view of the cyst showing stratified squamous epithelium without a granular layer and trichilemmal type of keratin in the lumen.

The second most common cyst found in our study was the trichilemmal cyst (also known as the pilar cyst) accounting for 15.1% with scalp as the most common site. These findings are concordant with findings reported in the literature [2,10]. Female predilection was noted with the majority of the cases presenting in the fourth decade of life. Histologically, trichilemmal cysts showed benign stratified squamous epithelium without granular layer, abrupt trichilemmal type of keratinization, and associated inflammation in ruptured cases as shown in Figures 2A, 2B [3,9].

**Figure 3:**
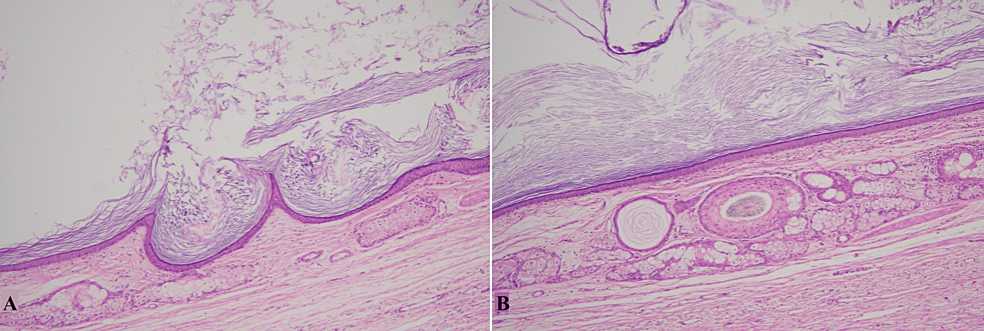
Dermoid cyst A and B: Cyst with squamous lining, flaky keratin in the lumen, and pilosebaceous units in the wall.

The dermoid cyst was the third most prevalent cyst in our study accounting for 6.3% which is discordant with the findings of Inbasekaran et al., and Singh et al. [1,9]. It showed female predominance, with eye as the most common site and the majority of cases presenting in the second and third decade of life. Histologically, dermoid cysts showed variable components for example different types of epithelial lining, skin adnexal structures in the wall, keratinous debris, hair shafts, inflammation, and giant cell reaction, as shown in Figures 3A, 3B. In our study, the most common type of epithelial lining documented in the dermoid cyst was stratified squamous which is concordant with findings in the literature.

**Figure 4:**
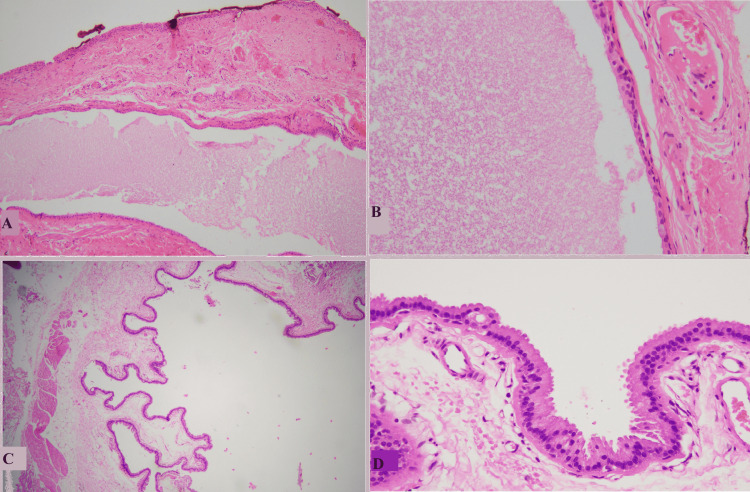
Eccrine and apocrine hidrocystomas A: Low-power view of eccrine hidrocystoma. B: High-power view of eccrine hidrocystoma showing flattened to cuboidal lining. C: Low-power view of apocrine hidrocystoma. D: High-power view of apocrine hidrocystoma showing double lining with abundant cytoplasm and apical snouts

Hidrocystoma was the fourth most prevalent cyst accounting for 1.9%. Male prevalence was noted with the majority of the cases presenting in the fourth and fifth decade of life. Eye was the most common site of presentation for these cysts. Hidrocystomas arise from dermal sweat glands and have two morphological variants namely eccrine hidrocystoma and apocrine hidrocystoma [11-13]. Eccrine hidrocystomas showed flattened to cuboidal double lining with cells having scant cytoplasm as shown in Figures 4A, 4B. Apocrine hidrocystomas showed double lining with outer myoepithelial and inner epithelial cells having abundant eosinophilic cytoplasm and apical snouts as shown in Figures 4C, 4D [12-15].

**Figure 5:**
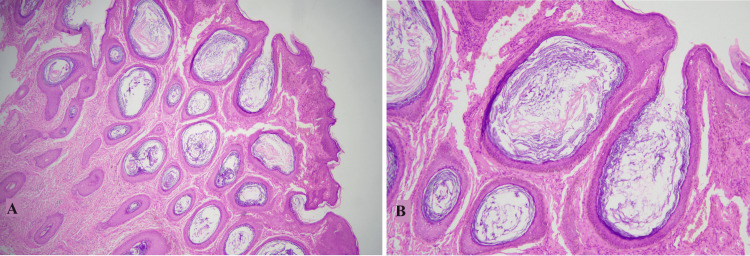
Comedone A: Low-power view of comedones representing multiple small keratin containing cysts. B: High-power view showing closed and open comedones with squamous lining and keratin in the lumen.

Comedones represent a form of acne vulgaris which are multiple small open or closed cystic lesions lined by stratified squamous epithelium with luminal keratinous debris as shown in Figures 5A, 5B [16,17]. In this study, a striking male predominance was noted for comedones and more than half of the cases were reported in the second decade of life with face as the most common site. These results are concordant with findings in the literature [17-19].

**Figure 6:**
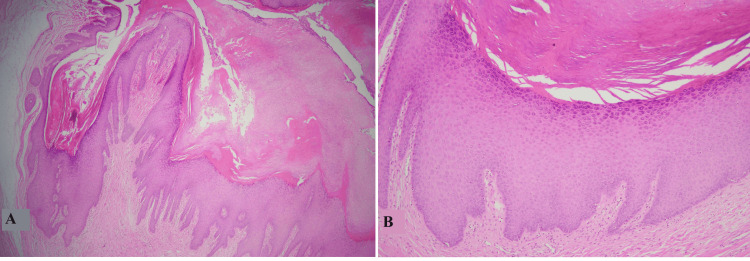
Verrucous cyst A: Low-power view of the cyst showing hyperplastic stratified squamous lining with keratin in the lumen B: High-power view of the cyst showing hyperplastic stratified squamous epithelium with hypergranulosis and keratin in the lumen

Verrucous cysts showed hyperplastic stratified squamous epithelium with hypergranulosis, prominent keratohyalin granules, squamous eddies within the epithelial lining, and prominent luminal keratinous debris as shown in Figures 6A, 6B [20-22]. These cysts are found to be associated with HPV infection [20,21,23,24]. The most common site for verrucous cysts was scalp in our study which is discordant while the age range in our study was concordant with the findings of Soyer et al. [24].

**Figure 7:**
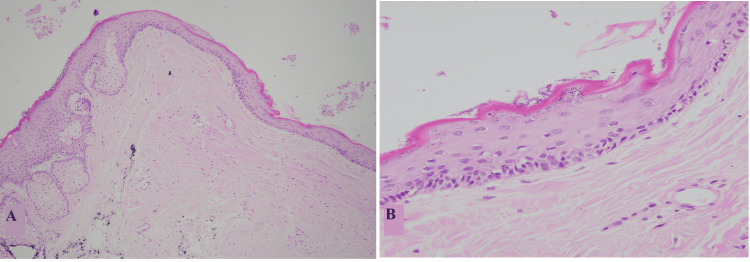
Steatocystoma A: Low-power view of the cyst showing stratified squamous epithelium, sebaceous units in the wall, and empty lumen. B: High-power view of the cyst showing stratified squamous epithelium without a granular layer and prominent surface corrugations.

Steatocystomas showed cysts lined by stratified squamous epithelium with prominent surface corrugations and an absent granular layer along with sebaceous units within the cyst wall. These cysts show empty lumen on histology as shown in Figures 7A, 7B [25,26]. The most common site for steatocystoma in our study was face which is discordant with the findings of Cho et al. [27,28].

**Figure 8:**
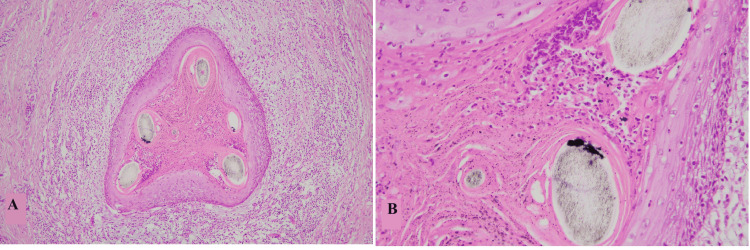
Vellus hair cyst A: Low-power view of the cyst showing stratified squamous epithelium and multiple vellus hair admixed with luminal keratin. B: High-power view of the cyst showing stratified squamous epithelium, vellus hair shafts, and keratinous debris in the lumen.

Vellus hair cysts represented small multiple dermal-based cysts with stratified squamous epithelium and keratinous luminal debris along with multiple hair shafts in the lumen as shown in Figures 8A, 8B [28-30]. The most common site for vellus hair cysts in our study was face which is discordant with findings of Anand et al. [31].

**Figure 9:**
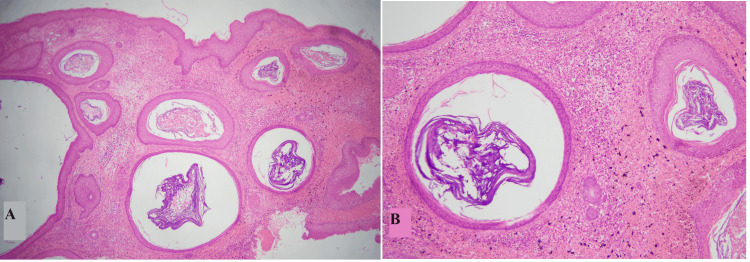
Milia A: Low-power view showing multiple small keratinous cysts. B: High-power view of the cyst showing stratified squamous epithelium and luminal keratin.

Milia were small multiple dermal-based cysts lined by squamous epithelium and contained keratin in the lumina as shown in Figures 9A, 9B. These arise from hair follicles and have characteristic clinical presentation [32,33]. Milia mostly present in newborns; however, they sometimes appear in adults. In our study, all cases were presented in the adult age group as these lesions are rarely biopsied in newborns [34,35]. The most common site reported in our study was face for milia which is concordant with the findings of Pastukhova et al. [36].

Hybrid cysts represented a combination of any of the cyst types described above. All three hybrid cysts in our study were a combination of epidermal inclusion cysts and trichilemmal cysts which is the most common combination described in the literature [37].

Limitations

This was a single-center study with a limited sample size as no collaboration with central registries can be established. 

## Conclusions

Benign cutaneous cysts have a broad morphological spectrum with a wide age range with an average age of 38 years. Epidermal inclusion cysts, trichilemmal cysts, dermoid cysts, and hidrocystoma account for the four most common types of benign cutaneous cysts. Other cutaneous cyst types were uncommon. Overall female gender predominance was noted for epidermal inclusion cysts, trichilemmal cysts, and dermoid cysts, while male predilection was noted for other types of cysts. The overall majority of the cysts presented in the head and neck area. In conclusion, as these lesions have a broad morphological spectrum and affect a wide age range, histological assessment is mandatory for correct categorization and documentation of the benign nature of these lesions.
